# Parametric study and process modeling for metronidazole removal by rhombic dodecahedron ZIF-67 crystals

**DOI:** 10.1038/s41598-023-41724-y

**Published:** 2023-09-05

**Authors:** Sajad Mazloomi, Ali Amarloei, Faeze Gholami, Gholam Ali Haghighat, Gagik Badalians Gholikandi, Heshmatollah Nourmoradi, Ali Akbar Mohammadi, Mehdi Fattahi, Binh Nguyen Le

**Affiliations:** 1 Biotechnology and Medicinal Plants Research Center, Faculty of Medicine, Ilam University of Medical Sciences, Ilam, Iran; 2 Department of Environmental Health Engineering, School of Public Health, Ilam University of Medical Sciences, Ilam, Iran; 3 Health and Environment Research Center, Ilam University of Medical Sciences, Ilam, Iran; 4 Department of Environmental Health Engineering, School of Public Health, Iran University of Medical Sciences, Tehran, Iran; 5 Faculty of Civil, Water and Environmental Engineering, Shahid Beheshti University, Tehran, Iran; 6 Department of Environmental Health Engineering, School of Health, Jiroft University of Medical Sciences, Jiroft, Iran; 7 Department of Environmental Health Engineering, Neyshabur University of Medical Sciences, Neyshabur, Iran; 8 Institute of Research and Development, Duy Tan University, Da Nang, Vietnam; 9 School of Engineering &Technology, Duy Tan University, Da Nang, Vietnam

**Keywords:** Environmental sciences, Chemistry

## Abstract

Metronidazole (MNZ) is an extensively used antibiotic against bacterial infections for humans and farm animals. Prevention of antibiotics discharge is essential to prevent adverse environmental and health impacts. A member of metal–organic frameworks, zeolite imidazole framework-67 with cobalt sulfate precursor (ZIF-67-SO_4_) and exceptional physio-chemical properties was prepared via room temperature precipitation to adsorb MNZ. The study framework was designed by Box–Behnken Design to evaluate the effect of pH, ZIF-67-SO_4_ dose, and contact time on adsorption efficiency. The polynomial model fitted the adsorption system indicated the optimal condition for 97% MNZ removal occurs at pH = 7, adsorbent dosage = 1 g/L, and mixing time = 60 min. The model also revealed that the removal increased with contact time and decreased at strong pH. Equilibrium and kinetic study also indicated the adsorption of MNZ followed the intra-particle diffusion model and the Langmuir isotherm model with a qmax = 63.03 mg/g. The insignificant loss in removal efficacy in use-reuse adsorption cycles reflected the practical viability of ZIF-67-SO_4_.

## Introduction

Metal–organic frameworks (MOFs) are a new class of materials consisting of metal ions or clusters coordinated with organic ligands to form one-, two-, or three-dimensional structures^1^. The open frameworks in MOFs made them unique materials with extraordinary properties such as permanent porosity, stable framework, enormous surface area, and pore volume^2^. MOFs attracted significant attention in recent years due to their unique properties and potential applications in various fields^3^. MOFs have a high surface area and tunable pore size, which makes them attractive for gas storage and separation. They have also been used for catalysis, drug delivery, sensing and energy storage applications^4^.

Zeolitic imidazolate frameworks (ZIFs) are a type of MOF that have been extensively studied due to their high thermal and chemical stability, tunable pore size, and high catalytic activity. ZIF-67 is a cobalt-based MOF that has been used for various applications such as adsorption, separation, electrochemistry, and catalysis. It also has been used as adsorbent for extracting herbicides in aqueous samples in the solid-phase microextraction (SPME) technique^5^, and as a catalyst for the condensation reaction of benzaldehyde with nitromethane. The structural, and electrical properties of ZIF-67 made it interesting material for the synthesis of catalysts and heterojunctions^6–8^.

The morphological evolution of Co-MOF-based ZIF-67 was investigated in the literature by changing the reaction conditions, including the cobalt source, type of solvent, aging time, crystallization temperature, and organic linker for the cobalt ions ^6^. ZIF-67 prepared by cobalt sulfate has interesting physical and chemical properties that make it an excellent candidate for various applications such as adsorption of volatile organic compounds (VOCs), separation, supercapacitors, and catalysis.

The choice of metal source is critical in the synthesis of Zeolitic Imidazolate Frameworks (ZIFs) as it affects the morphology and physicochemical properties of the product^9^. For instance, using Co(NO_3_)_2_ as a cobalt source results in small-sized and agglomerated ZIF-67 crystals. On the other hand, small-sized rhombic dodecahedron crystals were obtained when CoCl_2_ was introduced in the synthesis solution. Likewise, large-sized crystals of rhombic dodecahedron structure were obtained by the application of CoSO_4_ and Co(OAc) salts^10^. The flexible and engineerable structure of ZIFs and other MOFs make them attractive target materials in environmental remediation such as water treatment.

Water is an indispensable environmental resource for all forms of life. However, the contamination of water has become a major environmental hazard for humans and other living species. The extensive use of antibiotics has been associated with the contamination of various compartments of the environment such as surface water, groundwater, drinking water, municipal sewage, soil, vegetables, and sludge^11^. The release of antibiotics by antibiotic manufacturers discharges, animal farming, and hospital wastewater is a significant concern for human health and the environment^12,13^. Many antibiotics are classified as refractory chemicals with potential mutagenicity, and carcinogenicity^14^. In particular, metronidazole (MNZ) is a widely used antibiotic against bacterial infections in humans and farm animals. The conventional treatment techniques are inefficient in abating MNZ as it is highly soluble in water and also has a very robust chemical structure. MNZ can accumulate in the human body and the environment due to its recalcitrant nature. Encephalopathy, seizures, cerebellar, and spinal cord damage are among the harmful effects of MNZ ingested by drinking water^15,16^.

Of different pollutant removal techniques, adsorption is nominated as a convenient, economical, and friendly practice. This is due to the simple design and operation of the system, flexibility of adsorbent materials for different contaminants, reusability of materials, and efficiency of the process special in low levels of pollutants. Despite drawbacks such as requiring adsorbent regeneration, adsorption is still an advantageous treatment technique that produces minimal sludge and polluted streams that are hard to handle^17,18^.

The adsorption of metronidazole from aqueous media has been studied to reduce its pollution in water bodies. Kalhorizadeh et al.^19^ conducted a study on MNZ removal using two model metal–organic frameworks (MOFs), namely NH 2-MIL-101-Fe and HKUST-1. The authors concluded that both MOFs are effective in removing MNZ and NH2-MIL-101-Fe was identified as a superior adsorbent compared to HKUST-1.

Seo et al. reported MIL-101 (Cr) modified with urea or melamine for improved removal of nitroimidazole from water^20^. In another study, eight water-stable MOFs were screened for the removal of MNZ from water. The authors reported that two isostructural Zr (IV)-MOFs (UiO-66 and UiO-66-NH_2_) have a promising adsorption efficacy for MNZ^21^. In another study, ZIF-8 was synthesized and used for the removal of MNZ from water. The authors reported the MNZ removal in optimal condition predicted by the quadratic model reached to a high percentage of ~ 94%^22^.

Due to the unique properties i.e., uniform crystals, structural robustness, facile and environmentally friendly preparation method in aqueous medium, and high-yield production, ZIF-67-SO_4_ was investigated in this study as a viable material for MNZ removal. The key physicochemical parameters such as contact time, adsorbent mass, pH, and MNZ concentration were experimented to understand their impacts on the sorption system. Box–Behnken design (BBD), a particular technique of response surface methodology (RSM) that uses a statistical approach to design the experiments in three parametric levels was used to develop a mathematical model. The model provided an in-depth view on the process, and a tool to optimize the process for the highest removal, and also to describe the interactions of operating variables on MNZ adsorption.

## Materials and methods

### Chemicals

Zeolitic imidazolate framework-67 (ZIF-67) is composed of cobalt ions and 2-Methylimidazole (HMIN) linkers. Cobalt sulfate (CoSO_4_) was used as a source of metal in ZIF-67. All chemicals used in the synthesis of ZIF-67 and preparation of metronidazole (MNZ) solution were analytical grade and purchased from Merck or Sigma-Aldrich. Deionized (DI) water with an electrical conductivity of less than 0.5 µs/cm was used throughout the study for the experiments. Acetonitrile (ACN) and DI with HPLC grade were used for measuring MNZ.

### Adsorbent preparation

ZIF-67-SO_4_ was synthesized at room temperature using a simple precipitation technique described earlier^23^. 0.281 g of CoSO_4_ was added to a 10 mL solution of deionized water to prepare the metal precursor. A second solution was prepared by adding 1.642 g HMIM to 10 mL DI. To obtain ZIF-67, the two solutions were mixed and stirred for 30 min to complete the formation of crystals. After crystals formed, the precipitates were separated by centrifugation at 5000 rpm, washed with deionized water, and dried at 80 °C overnight.

### Adsorption experiments

The adsorption efficacy of ZIF-67-SO_4_ was evaluated in a batch mode operation system by MNZ (25 mg/L) as the contaminant of interest. The samples were mixed using a magnet stirrer at 250 rpm. The temperature (23 ± 1 °C) and mixing speed were not changed throughout the experiments. MNZ concentration was determined by high-performance liquid chromatography (HPLC; Knauer smartline, Germany) equipped with a C18 column and UV–VIS detector at 320 nm. For the operation of HPLC, the mobile phase was ACN: WATER in the ratio of 60:40. The experiments were performed twice and the average values were used for data analysis.

### BBD modeling and optimization

Response Surface Methodology (RSM) was used to study the effect of operating factors. RSM is a set of statistical and mathematical methods that are used to explore the relationships between several explanatory variables and to optimize one or more response variables result in the desired output. Box–Behnken Design (BBD) is a particular type of RSM that is specially designed to fit a second-order model on the data^24^. BBD design has the advantage of being highly accurate, easy to implement, and requiring fewer trials compared to other RSM and also one-factor-at-a-time techniques. The variables and the level of each factor in this study are shown in Table 1.

**Table 1:** The variables and the level of each factor in this study.

Factor	Code	Variable level		
		− 1	0	+ 1
ZIF-67-SO_4_ (g/L)	A	0.25	0.625	1
Mixing time (min)	B	15	37.5	60
pH	C	4	7	10

After generating the experimental matrix in Table 2, the sorption experiments were accomplished as ordered in the table and the final MNZ concentration was determined in clear centrifuged solutions. The adsorption removal efficiency for ZIF-67-SO_4_ and the capacity of adsorbent (q) for MNZ were responses in this study and calculated by the following equations:

1 $${\text{Adsorption removal }}\left( {\text{\% }} \right) = \frac{{\left( {{\text{C}}_{{\text{i}}} - {\text{C}}_{{\text{t}}} } \right)}}{{{\text{C}}_{{\text{i}}} }}{ } \times { }100$$

2 $$q_{t} \left( {{\text{mg}}/{\text{g}}} \right) = \frac{{\left( {{\text{C}}_{{\text{i}}} - {\text{C}}_{{\text{t}}} } \right) \times {\text{V}}}}{{\text{m}}}$$

3 $${\text{q}}_{e} \left( {{\text{mg}}/{\text{g}}} \right) = \frac{{\left( {{\text{C}}_{{\text{i}}} - {\text{C}}_{{\text{f}}} } \right) \times {\text{V}}}}{{\text{m}}}$$

where C_i_, C_t_, and C_f_ are the concentration of MNZ (mg/L) in the solution before adsorption begins, at any specific time, and the equilibrium, respectively. In the equations, m is the ZIF-67-SO_4_ mass (g) and V is the volume of the solution (L), respectively.

**Table 2:** The experimental matrix and corresponding responses for MNZ removal by ZIF-67-SO_4_.

Order	Adsorbent dose	Mixing time	pH	Removal
	g/L	min	-	%
1	1	37.5	4	69
2	0.25	37.5	4	48
3	0.625	60	4	72
4	0.625	15	4	54.8
5	0.25	60	7	76
6	1	60	7	97
7	0.625	37.5	7	94.6
8	0.625	37.5	7	91.6
9	1	15	7	76.9
10	0.25	15	7	68
11	0.625	37.5	7	92.6
12	0.25	37.5	10	61.6
13	1	37.5	10	86.3
14	0.625	60	10	78.6
15	0.625	15	10	73

The data obtained experimentally were analyzed by Analysis of Variance (ANOVA) to set a polynomial equation describe the process response (Y = MNZ removal) as a function of operating factors i.e., X_i_ and X_j_:

4 $${\text{Y}} = {\upbeta }_{0} + \mathop \sum \limits_{{{\text{i}} = 1}}^{{\text{k}}} {\upbeta }_{i} {\text{X}}_{{\text{i}}} + \mathop \sum \limits_{{{\text{i}} = 1}}^{{\text{k}}} {\upbeta }_{ii} {\text{X}}_{{\text{i}}}^{2} + \mathop \sum \limits_{{{\text{i}} = 1}}^{{{\text{k}} - 1}} 1\mathop \sum \limits_{j - 1}^{{\text{k}}} {\upbeta }_{ij} {\text{X}}_{{\text{i}}} {\text{X}}_{{\text{j}}} + {\upvarepsilon }$$

where β_0_ is a constant value, β_i_, β_ii_, and β_ij_ are the coefficients for linear, second-order, and interaction effects, and $\upvarepsilon$ is the error constant for the model.

Once the model is developed, optimization of the polynomial equation accomplished to determine the best level for each variable in which the highest MNZ adsorption happens. The optimal condition was then validated through the additional experiments.

### Isotherm and kinetic studies

Isotherm and kinetic studies are since they assist to realize the behavior of the adsorbent and the mechanism governing the adsorption. Isotherm models mirror the relationship between the mass of adsorbate on adsorbent and its concentration in solution at equilibrium. Kinetic models, on the other hand, are extensively used to determine the rate of adsorption which is an important factor in the economy of the process. The models are crucial in the optimal design and operation of the real treatment unit. In this study, kinetic and isotherm experiments performed in the optimal condition described in optimization section. For the equilibrium study, the concentration of MNZ was studied in the range of 5–50 mg/L.

Langmuir model is a theoretical equation that explains the adsorption of gases onto a solid surface and was proposed in 1916. The Langmuir isotherm assumes that adsorption is limited to a monolayer, the adsorbent surface is homogeneous, and all sorption sites are energetically identical. The Freundlich isotherm, on the other hand, is a mathematical model that describes the multilayer adsorption of a solute onto a heterogenous adsorbent surface^25^.

The Temkin isotherm is another popular model in environmental studies. The assumption behind the development of the model is that the adsorption temperature of all molecules decreases as the surface of the adsorbent becomes more covered^26^. The empirical Redlich–Peterson (R–P) isotherm is commonly used model to describe adsorption in microporous materials.

The pseudo-first-order (PFO) and pseudo-second-order (PSO) kinetic models are common kinetic models to describe the rate of adsorption. The former model describes the adsorption system as proportional to the number of free-binding sites on the surface. The PSO, otherwise, attributed the adsorbate attachment to the surface by the chemical bonding^27^. The intra-particle diffusion (IPD) model, describes the pollutant migration process from liquid balk to the adsorbent pores and assumed it as the rate-limiting step in adsorption^28^.The nonlinear forms of isotherm models are provided in Table S1^29^.

## Results and discussion

### ZIF-67-SO_4_ characteristics

**Figure 1:**
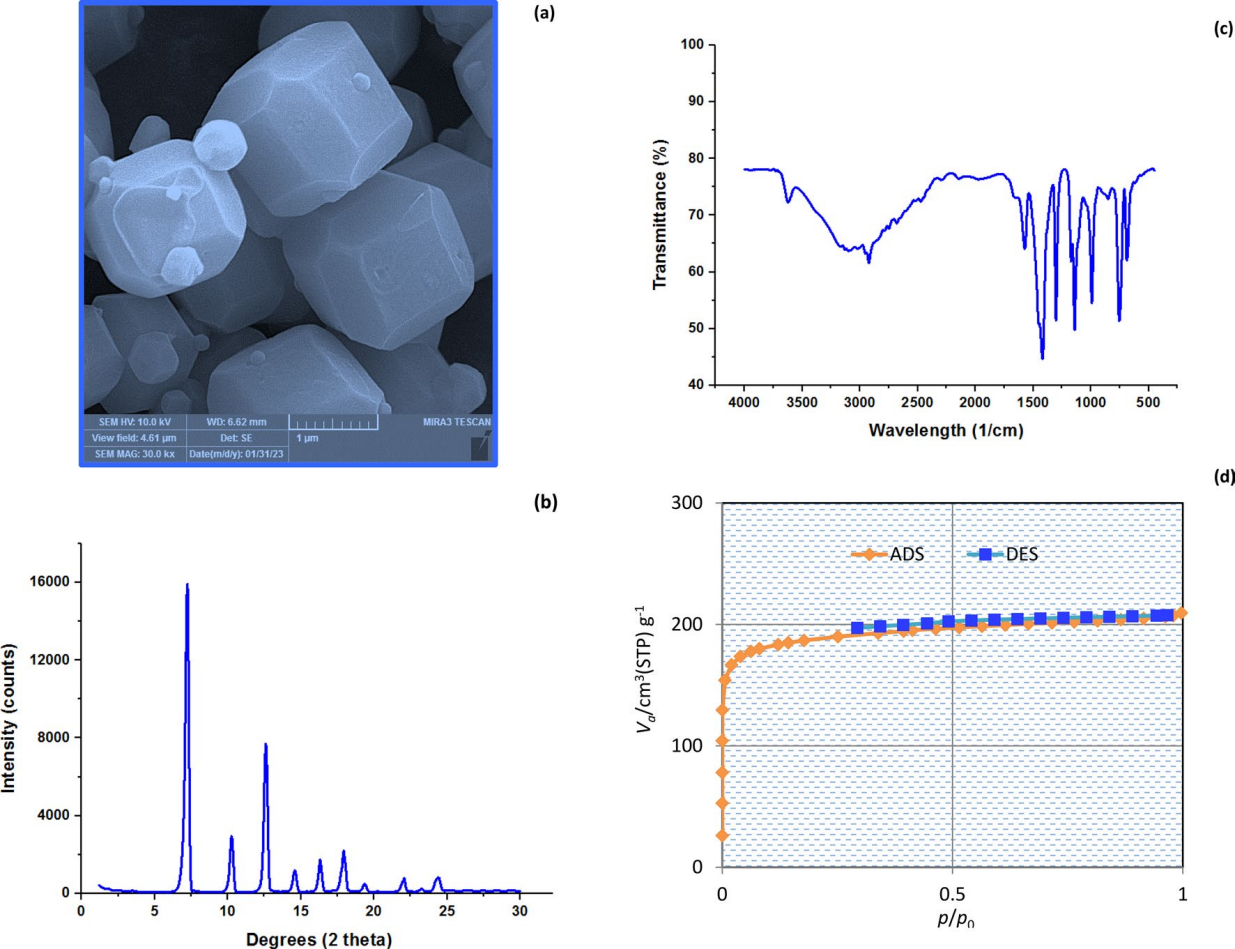
The structural properties of ZIF-67-SO_4_, FESEM image (**a**), XRD pattern, FTIR spectrum, and N_2_ adsorption–desorption.

The as-synthesized ZIF-67-SO_4_ was analyzed by scanning electron microscopy (FE-SEM, MIRA3 TESCAN, Czech Republic) to reveal information about the texture morphology and crystalline structure. X-ray diffraction (XRD; Unisantis S.A, XMD300 model, Geneva, Switzerland) using Cu-kα beam was also used for phase identification of crystalline ZIF-67-SO_4_. Fourier-transform infrared spectroscopy (FTIR; Thermo Nicolet, Avatar 370), and N_2_ adsorption–desorption (BELSORP-mini-II BEL Japan, Inc.) also performed to identify the functional groups, and pore volume/ specific surface area, respectively. Figure 1a shows the SEM image of ZIF-67-SO_4_ crystals that have the uniform truncated rhombic dodecahedral framework. The XRD pattern of ZIF-67-SO_4_ shown in Fig. 1b shows the major characteristic peaks correspond to the crystallographic planes of the ZIF-67-SO_4_ at 7.6, 10.6, 13.1, and 17.9 degrees of 2θ assigned to the (011), (002), (112), and (222) planes, respectively^30^.The ZIF-67-SO_4_ FTIR spectrum in Fig. 1c displays peaks at 3404 cm^−1^ for N–H stretching, 3128 cm^−1^ and 2907 cm^−1^ for C–H stretching from the methyl group on the imidazole ring, a peak at 1572 cm^−1^ for CN stretching, peaks from 1428 to 667 cm^−1^ due to the stretching of the imidazole ring, and a peak at 414 cm^−1^ due to Co–N stretching. These characteristic peaks confirm the bond developed between cobalt and the linker^31^. N_2_ adsorption desorption analysis performed and indicated the porous nature of the adsorbent structure. The SSA, total pore volume, and average pore diameter of ZIF-67-SO_4_ crystals based on the BET model in Fig. 1d were 737.5 m^2^/g, 0.322 m^3^/g, and 1.75 nm, respectively.

### MNZ modeling and optimization

BBD design is an interesting statistical approach that conducted to fit a non-linear model on system performance as a function of study variables. In BBD, the number of experimental runs is determined by the following formula:

5 $${\text{N}}\, = \,{2}^{{ {\text{K}}}} \, + \,{2} {\text{K}}\, + \,{1}$$

In Eq. 5, N is the number of experimental runs, and K is the number of variables. Table 2 presents the study matrix consisting of 15 runs, and responses for each experiment. As seen, the removal efficiencies ranged from 48 to 97%, which are the lowest and highest values recorded experimentally.

The experimental efficiencies were undergoing an ANOVA statistical analysis to fit a polynomial model. The result of the quadratic model fitted to the experimental data is presented in Table 3.

**Table 3:** ANOVA for the quadratic model fitted to MNZ removal by ZIF-67-SO_4_.

Source	Sum of squares	DF	Mean square	F-value	*p* value
Model	2954.31	9	328.26	37.27	0.0005
A-dose	714.42	1	714.42	81.12	0.0003
B-time	323.85	1	323.85	36.77	0.0018
C-pH	387.81	1	387.81	44.04	0.0012
AB	36.60	1	36.60	4.16	0.0970
AC	3.42	1	3.42	0.3886	0.5604
BC	33.64	1	33.64	3.82	0.1081
A^**2**^	261.56	1	261.56	29.70	0.0028
B^**2**^	93.85	1	93.85	10.66	0.0223
C^**2**^	1235.39	1	1235.39	140.28	< 0.0001
Residual	44.03	5	8.81		
Lack of Fit	39.37	3	13.12	5.62	0.1547
Pure Error	4.67	2	2.33		
Cor Total	2998.34	14			
R^**2**^	0.9853				
Adjusted R^2^	0.9589				
Adeq Precision	19.6343				

The F-value is an important statistical value that is used to check whether the null hypothesis should be rejected or not. The Model F-value of 37.27 implies it is significant and there is only a 0.05% chance that an F-value this large could occur due to the noise. If the p-value is less than 0.05, it indicates that the model terms in Table 3 are significant. In this case, A, B, C, A^2^, B^2^, C^2^ are significant model terms. The Lack of Fit (LOF) F-value is also a significant statistical value that reflects whether the LOF is significant relative to the pure error. For the developed model, the LOF F-value of 5.62 implies that the Lack of Fit is not significant. The Predicted R^2^ of 0.7864 is in rational agreement (within ± 0.2) with the R^2^_Adj_ of 0.9589. While R^2^ measures how well a regression model fits the data, the Predicted R^2^ is calculated by a subdivision of the data to predict the residual data. The Adjusted R^2^ is a revised form of R^2^ that used to compare different models with different numbers of predictors as the parameters adjusts for the number of predictors. Another statistical indicator, Adeq Precision, measures the ratio of signal to noise. The value of 19.634 is above the minimum desirable value of 4 and indicates an adequate signal. A polynomial equation was developed based on quadratic model coefficients calculated for coded factors in Table 4.

**Table 4:** Coefficients calculated for the quadratic model based on the coded factors.

Factor	Coefficient Estimate	df	Standard Error	95% CI Low	95% CI High	VIF
Intercept	92.93	1	1.71	88.53	97.34	
A-dose	9.45	1	1.05	6.75	12.15	1.0000
B-time	6.36	1	1.05	3.67	9.06	1.0000
C-pH	6.96	1	1.05	4.27	9.66	1.0000
AB	3.03	1	1.48	− 0.7893	6.84	1.0000
AC	0.9250	1	1.48	− 2.89	4.74	1.0000
BC	− 2.90	1	1.48	− 6.71	0.9143	1.0000
A^2^	− 8.42	1	1.54	− 12.39	− 4.45	1.01
B^2^	− 5.04	1	1.54	− 9.01	− 1.07	1.01
C^2^	− 18.29	1	1.54	− 22.26	− 14.32	1.01

The final model equation based on code, and actual values for the variables are presented in Eqs. (6) and (7), respectively.

6 $$\begin{aligned} {\text{MNZ removal }}\left( \% \right) = & + \,{92}.{939}.{\text{45A}}\, + \,{6}.{\text{36B}}\, + \,{6}.{\text{96C}}\, + \,{3}.0{\text{3AB}} \\ & \quad + 0.{925}0{\text{AC}} - {2}.{9}0{\text{BC}} - { 8}.{\text{42 A}}^{{2}} - {5}.0{\text{4 B}}^{{2}} - {18}.{\text{29 C}}^{{2}} \\ \end{aligned}$$

7 $$\begin{aligned} {\text{MNZ removal }}\left( \% \right) = & - {85}.{91}\, + \,{8}0.{\text{81 dose}}\, + \,{1}.{\text{1 time}}\, + \,{31}.{\text{87 pH}}\, + \,0.{\text{35 dose}}\, \times \,{\text{time}} \\ & \quad + 0.{\text{82 dose}}\, \times \,{\text{pH }} - 0.0{\text{42 time}}\, \times \,{\text{pH }} - {59}.{\text{85 dose}}^{{2}} {-\!\!-}0.00{\text{9 time}}^{{2}} {-\!\!-}{2}.0{\text{3 pH}}^{{2}} \\ \end{aligned}$$

The Eq. 6 and Eq. 7 could be used as a tool to predict the MNZ removal for given levels of each factor. The coded equation (Eq. 6) provides a useful tool to identify the relative impact of each factor by comparing the factor coefficients. Accordingly, the adsorbent mass (A) has the highest coefficient in the model and hence the highest impact on the process. The equation in terms of actual factors (Eq. 7) can be used to predict the response for given levels of each factor in the original units.

### Effect of study variables

One essential part of the sorption study is analyzing how contaminants are removed as a function of operating variables. BBD design is a powerful tool to explain how independent factors and their interactions affect the process. ZIF-67-SO_4_ dose, pH, and mixing time were considered as independent variables for MNZ removal. 3D plots that were developed based on the Eq. 5. were used to illustrate how MNZ removal was changed by operating conditions.

**Figure 2:**
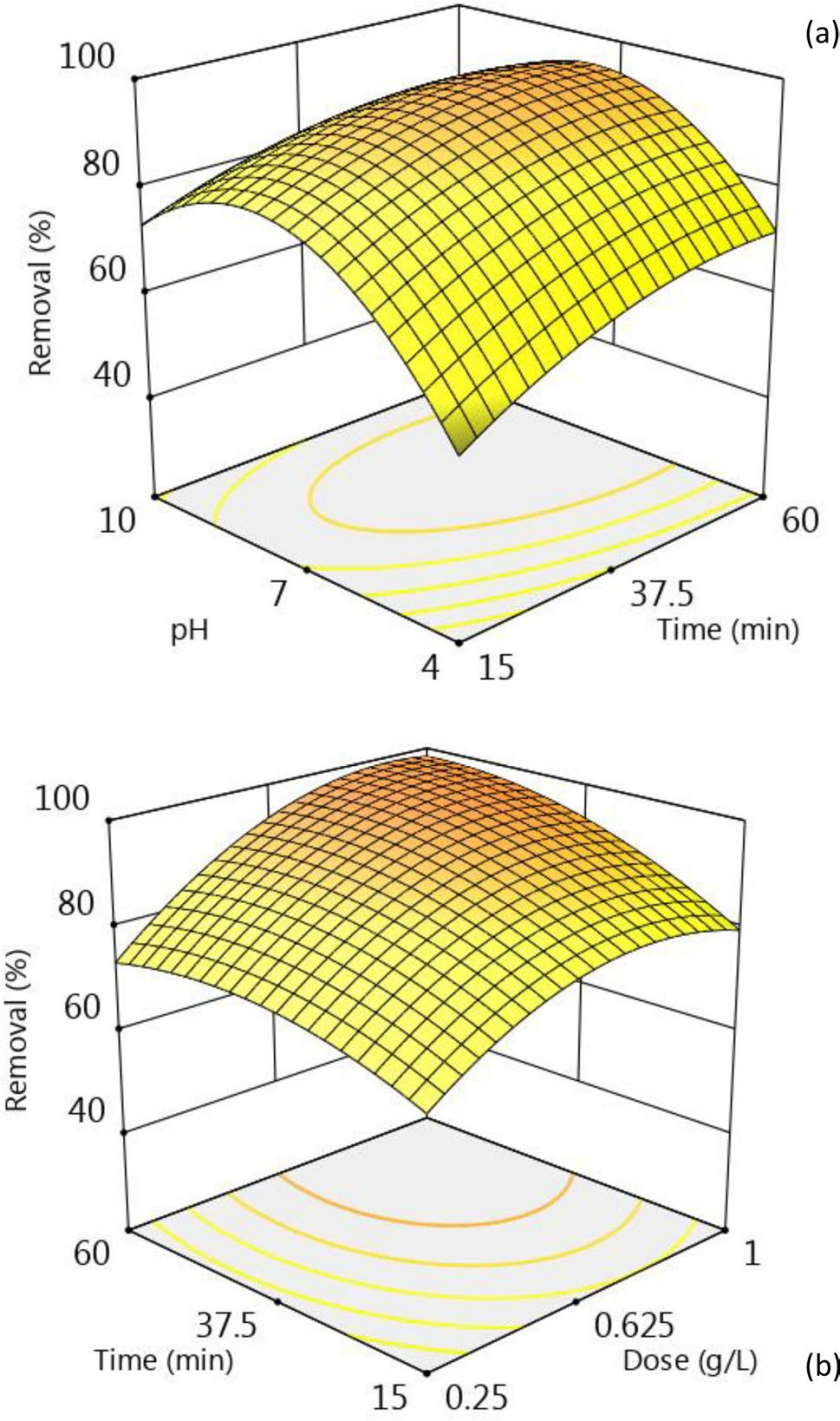
The 3D plots for the effect of independent variables on MNZ removal by ZIF-67-SO_4_.

Figure 2a shows the effect of pH and mixing time on the process. The pH of the solution can affect the charge of the adsorbent and ionic state of MNZ, so it has a significant effect on the process. The figure shows the antibiotic removal reached a maximum value at pH ~ 8. The pH_ZPC_ for the ZIF-67 was determined ~ 9.8 which means the crystal’s surface charge changed over different solution pH. Once the solution pH is < 9.8, the surface charge of the adsorbent becomes positive. A negative surface charge developed by increasing pH from 9.8. Changes in adsorbent surface charge directly impact MNZ removal by developing attraction or repellent electrostatic force. The pKa1 and pKa2 for MNZ as a weak base are 2.38 and 14.48, respectively. At pH > 4, MNZ has a positive charge as it is protonated in the solution. At pH 4–12, and pH > 12, MNZ is neutral and negatively charged since it is de-protonated by pH. The repelling electrostatic force by domination of positive surface charge and positive MNZ in the acidic environment, and negative surface charge of ZIF and anionic MNZ at alkaline conditions are responsible for removal drop. Furthermore, at the higher pH, the presence of OH^-^ also hinders MNZ ions to attach the adsorbent^29,30^. Lower MNZ adsorption in acidic condition, on the other hand, could be attributed to the net repulsive force that exists between the positive species MNZ^+^, H^+^, and ZIF-67^+^. Alamgir et al.^21^ studied MNZ removal and found a good adsorptive removal by UiO-66-NH2 in a wide range of pH from 4 to nearly 8. They attributed the adsorption to the development of electrostatic interaction and hydrogen bonding. Other studies also indicated that hydrogen bonding plays a critical role in the adsorption performance of MOFs due to the presence of functional groups^32^. Mixing time also is imperative variable in sorption systems as it provides the time required for contaminants to diffuse toward the adsorbent. Mixing time is also important for the economy of a treatment unit as it determines the volume required for the reactor. The figure shows a rapid uptake of ~ 50% MNZ at the initial 15 min witch gradually increased by mixing time to 60 min. Zeng et al.^33^ conducted a study on Doxycycline and Ciprofloxacin adsorption by biochar and found a rapid uptake of antibiotics in the first 30 min of the adsorption which gradually increased with time. Alsaedi et al.^34^ conducted another study that showed the maximum removal of Doxycycline by UiO-66 happened within 15 min. Zong et al.^35^ also reported a steep slope and a straight regression line in the first part of the tetracycline (TC) and ciprofloxacin (CIP) adsorption by Zr-MOFs, indicating a fast adsorption. The second part of the kinetic model had a gradual slope, indicating that the adsorption equilibrium changed slowly with time. The effect of ZIF-67-SO_4_ dose in the range of 0.25–1 g/L is presented in Fig. 2b. As seen, MNZ removal increased by the mass of adsorbent added to the solutions. Higher adsorbent mass result in a higher adsorption efficiency in many earlier studies. Elkady et al.^36^ prepared an environmentally friendly zirconium Bio-MOF for trimethoprim antibiotic. The parametric study of antibiotic removal showed increasing the MOF dose from 0.1 to 1.5 g/L increased the removal from 47.7 to 87.6%, respectively. In another study, MNZ removal by polypyrrole studied as a function of dose in the range of 0.05‒0.70 g/L. The authors reported an incremental removal by dose from about 60 to 89.55% when the dosage increased above 0.5 g/L. The optimal adsorbent dose of 0.5 g/L finally chosen based on the economic considerations^37^.

### Model optimization and validation:

**Figure 3:**
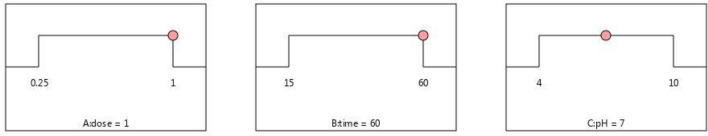
Graphical representation for the highest MNZ removal by ZIF-67-SO_4_.

Model optimization and validation are crucial in adsorption because they help to assure the model predict the response with adequate preciseness. Optimization also useful to identify the best operating condition for the best performance^38^. Herein, the model presented in Eq. 5 was solved for the highest MNZ removal and the graphical representation showed in Fig. 3. The highest MNZ removal of ~ 97% was achieved when pH = 7, adsorbent dosage = 1 g/L, and mixing time = 60 min. This condition was simulated and experimented to check the validity of the model optimization. The average antibiotic removal by three replications was 95.8 which is close that predicted by the model.

### Effect of MNZ concentration

**Figure 4:**
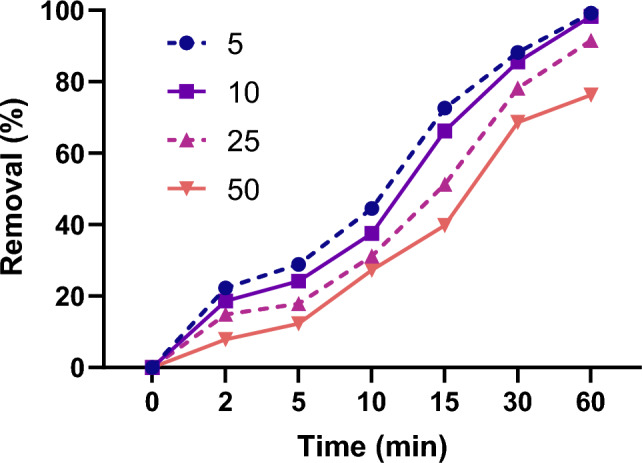
MNZ removal by ZIF-67-SO_4_ as a function of antibiotic concentration (ZIF-67-SO_4_: 1g/L, pH = 7).

Once the optimum levels of operating parameters determined, the process was studied as a function of MNZ concentration. Solutions of different antibiotic concentrations in the range of 5–50 mg/L were prepared and adsorption experimented in the presence of 1 g/L ZIF-67-SO_4_, and solution pH = 7. MNZ removal calculated at different time intervals and the results are presented in Fig. 4. As seen, ZIF-67-SO_4_ showed a significant adsorption property over a wide range of antibiotic concentrations, and up to 90% removal observed for the concentration of 25 mg/L. The graph also reveals the adsorption decreased from ~ 99 to ~ 76% when MNZ concentration increased in the studied range. A higher competition for limited adsorption sites on the surface is the possible cause of removal drop as also described in earlier works^9,39^.

### Effect of temperature

The temperature is an important variable is sorption of contaminants as it determines the diffusion rate of contaminants and also the adsorbent-adsorbate bonds. MNZ removal under optimum condition i.e., pH = 7, adsorbent dosage = 1 g/L, and mixing time = 60 min was studied as a function of temperature to realize the effect of solution temperature on the process. The study indicated that the MNZ removal decreased from ~ 96% in 23 ± 2 °C to about ~ 88% in 45 ± 2 °C that is an indication of exothermic adsorption. Different behavior in adsorption of MNZ reported for studied adsorbents. Sun et al. and Arbab et al. indicated the MNZ antibiotic adsorption onto biochar derived from Sugarcane Bagasse, and polypyrrole, respectively, is exothermic^37,40^. Nasseh et al.^41^ on the other hand, indicated the MNZ removal by FeNi_3_/SiO_2_/CuS magnetic nanocomposite increased from 37.73 to 65.15% as the temperature elevated in the studied range.

### Kinetic and isotherm studies

Kinetic and isotherm studies are important in adsorption because they provide valuable information about the adsorption process, the potential rate-limiting phase, and economy of treatment which can be used to optimize the design of adsorption systems and improve their efficiency^42^. The results of isotherm modeling in Table 5 shows the Sips model fit best on equilibrium data. Sips model is widely used to describe the adsorption of a solute onto a solid surface at a fixed temperature. This theorical model derived from the limiting behavior of the Langmuir and Freundlich isotherms and assumed that MNZ adsorption onto ZIF-67-SO_4_ occurred on localized specific sites. In addition, the model suggested that the there is no interaction between adsorbed MNZ molecules^43,44^.

**Table 5:** The isotherm parameters for MNZ removal by ZIF-67-SO_4_.

Langmuir	q_max_ (mg/g)	62.40
	K_L_ (L/mg)	1.64
	R^2^	0.99
	R^2^_adj_	0.98
	RSS	28.09
Freundlich	K_F_ (mg/g) (L/mg)^1/n^	30.33
	n	3.44
	R^2^	0.97
	R^2^_adj_	0.96
	RSS	67.27
Temkin	A_t_	23.56
	bT_1_	9.58
	R^2^	0.99
	R^2^_adj_	0.98
	RSS	19.67
Dubinin–Radushkevich	kRP (mg/g)	191.24
	aRP	4.29
	R^2^	0.99
	R^2^_adj_	0.99
	RSS	2.42
Sips	qms	71.06
	KS	1.02
	Ms	0.69
	R^2^	0.99
	R^2^_adj_	0.99
	RSS	1.33

The maximum monolayer adsorption capacity (qmax) of ZIF-67-SO_4_ for MNZ was 63.03 mg/g which is an essential tool to compare different adsorbents. Table 6 listed the qmax of ZIF-67-SO_4_ and other adsorbents studied against MNZ. As seen, ZIF-67-SO_4_ and other MOFs are generally promising materials with significant capacities for MNZ. A significant improvement also is attainable by preparing composites and modified forms of MOFs that indicated the versatile and engineerable nature of these emerging materials.

**Table 6:** Qmax of ZIF-67-SO_4_ and other adsorbents for MNZ.

MOF	Qmax (mg/g)	Reference
AgN/MOF-5	191.1	^45^
UiO-66-NH2	265.5	^21^
Chitosan-Modified Graphene Oxide	29.76	^46^
polypyrrole (PPy)	5.05	^37^
Urea-MIL-101	188	^20^
ZIF-8 Cu-ZIF-8	30 16	^22^
ZIF-67-SO_4_	62.40	This study

Table 7 listed the non-linear kinetic models and related parameters for MNZ removal by ZIF-67-SO_4_. As seen, the data fit to the Pseudo-first order (PFO), pseudo-second order (PSO), and intra-particle diffusion (IPD) models. The data also illustrated graphically and presented in Fig S1. As seen, the IPD model fit best with non-linear model. According to IPA, the adsorbate diffusion inside the pores proposes as the rate-limiting step in the overall adsorption process^47,48^.

**Table 7:** The kinetic parameters for MNZ removal by ZIF-67-SO_4_.

Kinetic model	parameter	value
	Concentration (mg/L)	25
Pseudo-first order		
${q}_{t}={q}_{e }{(1-e }^{-k1t}$)	q_e_ (mg/g)	25.36
	k_1_ (min^−1^)	0.05
	R^2^	0.97
	${\mathrm{R}}_{\mathrm{Adj}}^{2}$	0.97
	RSS	9.95
	χ^2^	1.99
Pseudo-second order		
${\mathrm{q}}_{\mathrm{t}}= \frac{{qe}^{2}{\mathrm{K}}_{2}\mathrm{t}}{1+ qe {\mathrm{k}}_{2 }t}$	q_e_ (mg/g)	33.38
	k_2_ (g/mg min)	0.001
	R^2^	0.98
	${\mathrm{R}}_{\mathrm{Adj}}^{2}$	0.97
	RSS	8.25
	χ^2^	1.65
Interparticle diffusion		
${q}_{t}={k}_{3 }\sqrt{t }+\mathrm{C}$	k_3_	3.24
	C	-0.06
	R^2^	0.98
	${\mathrm{R}}_{\mathrm{Adj}}^{2}$	0.98
	RSS	5.88
	χ^2^	1.17

### Reusability tests

**Figure 5:**
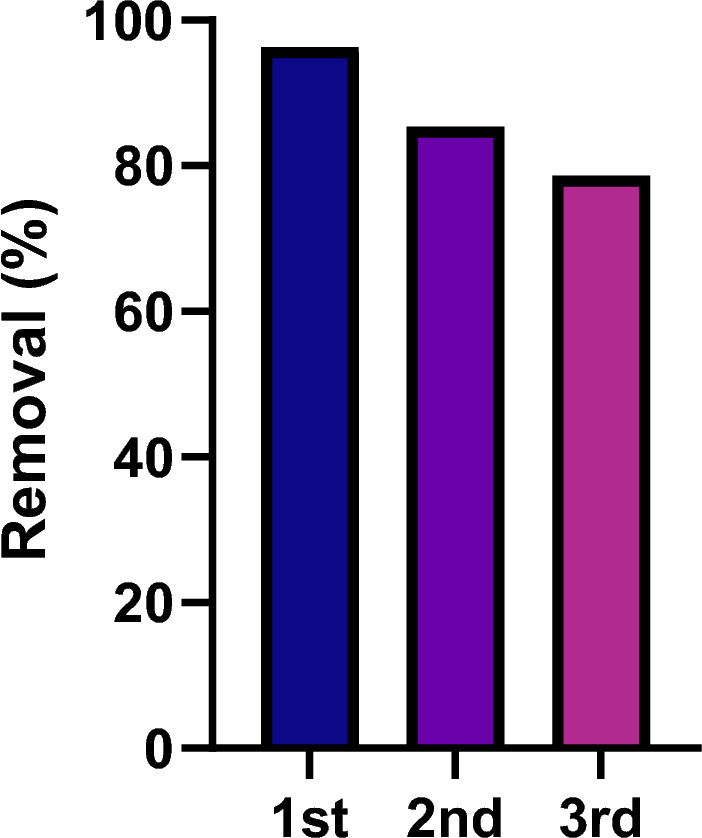
The reusability of ZIF-67-SO_4_ for use-reuse system (ZIF-67-SO_4_: 1g/L, pH = 7, mixing time = 60 min).

The regenerability of the adsorbents for adsorption–desorption cycles is a substantial feature that determines the economic and environmental viability of the sorption system. Hence, studying the adsorbent behavior at consecutive use-reuse series is of practical significance. Once pristine ZIF-67-SO_4_ was saturated with MNZ, desorption was accomplished by soaking the material in ETOH for 24 h while ETOH was replaced with the fresh solution every 4 h. ZIF-67-SO_4_ was finally washed thoroughly with deionized water and dried overnight for the next cycle. The reusability tests were accomplished at optimal conditions predicted by the model i.e., pH = 7, adsorbent dosage = 1 g/L, and mixing time = 60 min. Figure 5 indicated the MNZ removal decreased from 96.3% for pristine adsorbent to ~ 78.6% for the 3rd cycle. The insignificant loss in removal efficacy reflected the practical usability of ZIF-67-SO_4_.

### MNZ removal mechanism

ZIF-67-SO_4_ crystals are composed of metal cobalt ions joined by imidazolate rings similar to Si and Al atoms coordinated by oxygens in zeolites. The key physicochemical properties such as high available surface area, porosity, surface charge, and unidirectional flow of electrons are particular parameters responsible for metronidazole adsorption by ZIF-67-SO_4_. Developing hydrogen bonds between the nitrogen groups in MNZ and imidazolate rings and oxygen atoms, and also π-complexation between the cobalt ions and antibiotic rings are other mechanisms underlie the adsorption. In addition, the oxygen atom in the structure of MNZ is enough nucleophilic to bind with the metal species to form different coordination O–M configurations bonds.

## Conclusion

The presence of antibiotics in the environment threatens human health and there is a substantial demand to control their discharges by promising treatment techniques such as adsorption. Metal–organic frameworks (MOFs) received tremendous interest in recent years due to their substantial structural properties. A unique member of MOFs namely ZIF-67-SO_4_ employed as an adsorbent against the widely used antibiotic metronidazole (MNZ). An in-depth study of the process accomplished by Box–Behnken design (BBD) to reveal the effects of significant variables and their interactions. A quadratic model was developed to predict and to optimize the process efficacy. The model predicted the highest MNZ removal of 97% occurs at pH = 7, adsorbent dosage = 1 g/L, and mixing time = 60 min. MNZ removal was highest at near neutral pH and adsorbent mass was determined as the most influential variable in the process. Isotherm modeling indicated the MNZ adsorption followed the Langmuir isotherm with a qmax = 63.03 mg/g. ZIF-67-SO_4_ also studied in three consecutive use-reuse systems with ~ 17.7% loss in adsorption efficiency.

### Supplementary Information

Supplementary Information.

## Data Availability

The datasets generated and analyzed during the current study were available from the corresponding author on reasonable request.
