# Supplementary material for: Parametric study and process modeling for metronidazole removal by rhombic dodecahedron ZIF-67 crystals
Source: Sci Rep. 2023 Sep 5;13:14654. doi: 10.1038/s41598-023-41724-y (PMC10480145; doi:10.1038/s41598-023-41724-y)
Supplement: Supplementary file 1 — Supplementary Information. [file 41598_2023_41724_MOESM1_ESM.docx]

***Supplementary***

**Parametric study and process modeling for metronidazole removal by rhombic dodecahedron ZIF-67 crystals**

Table S1. The fitted isotherm models for MNZ sorption by ZIF-67-SO4

| Isotherm | Equation | Parameters |
| --- | --- | --- |
| Langmuir | $q_{e}= \frac{Q_{0}K_{L}C_{e}}{1+ K_{L}C_{e}}$ | C_e_= adsorbate equilibrium concentration (mg/L)  q_e_ = adsorption capacity at equilibrium (mg/g)  Q_0_= monolayer coverage capacity (mg/g)  K_L_= Langmuir isotherm constant (L/mg). |
| Freundlich | $q_{e}= K_{f}C_{e}^{1/n}$ | K_f_ = Freundlich isotherm constant(mg^1-(1/n)^ L^1/n^ g^-1^)  n = adsorption intensity |
| Temkin | $q_{e}= \frac{RT}{B}ln\left( A_{T}C_{e} \right)$ | A_T_ = the Temkin isotherm equilibrium binding constant (L/mg)  b_T_ = the Temkin isotherm constant  R = the universal gas constant (8.314 J/mol K)  T = the temperature (K)  B = the constant related to heat of sorption (J/mol) |
| Sips | $q_{e}=\frac{q_{m_{s}}K_{s}C_{e}^{m_{s}}}{1+K_{s}C_{e}^{m_{s}}}$ | $q_{m_{s}}$= Sips maximum adsorption capacity (mg/g)  K_S_ = Sips equilibrium constant ${(L/mg)}^{m_{s}}$  m_S_= Sips model exponent |
| Redlich-Peterson (R-P) | $q_{e}=\frac{K_{\mathrm{RP}}C_{e}}{1+a_{\mathrm{RP}}C_{e}^{g}}$ | K_RP_ = Redlich-Peterson isotherm constant(L/g)  a_RP_=Redlich-Peterson model constant(mg/L)^-g^  g = Redlich-Peterson model exponent |


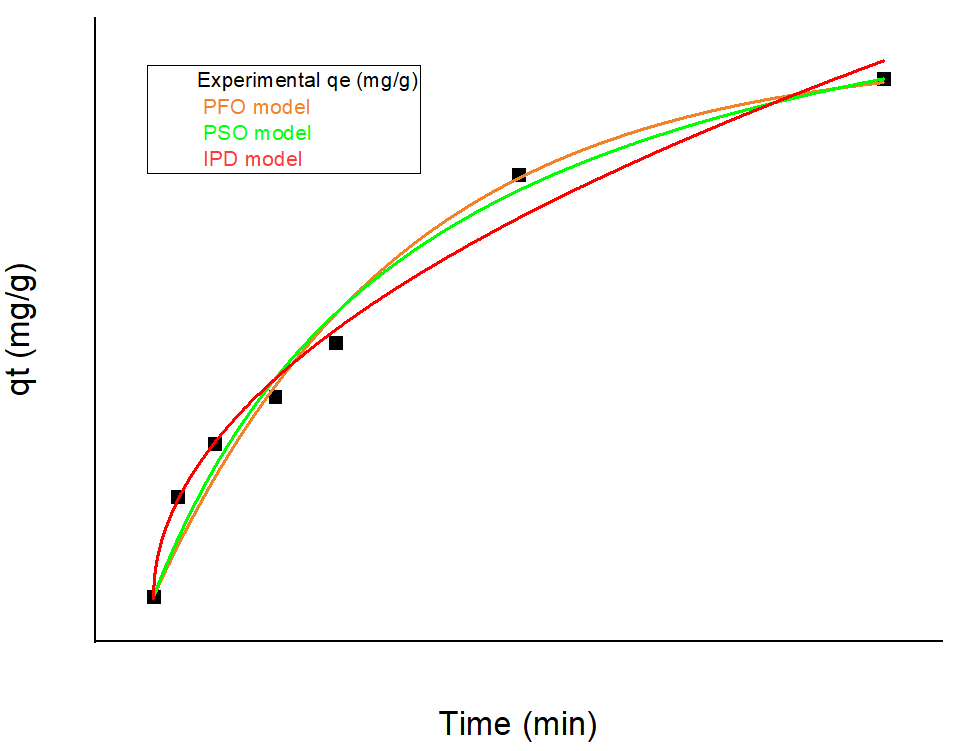


Fig. S1. Non-Linear kinetic models for MNZ removal by ZIF-67-SO4


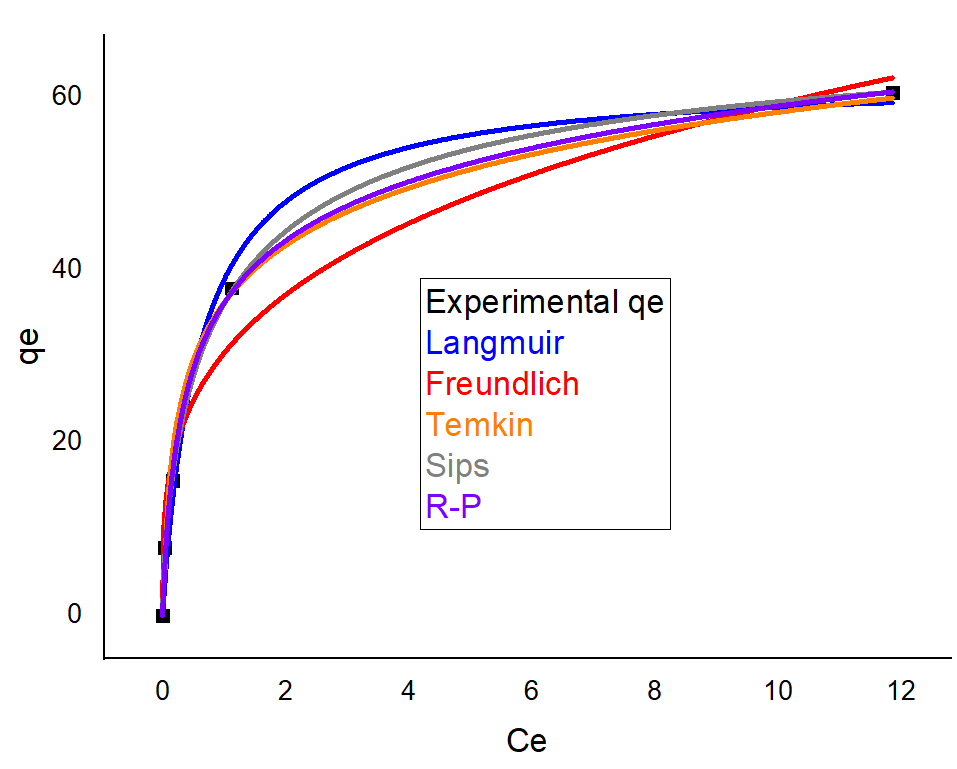


Fig. S2. Non-Linear isotherm models for MNZ removal by ZIF-67-SO4
